# The Effect of Hirodoid Cream on Ecchymosis and Edema around Eyes after Rhinoplasty

**DOI:** 10.29252/wjps.9.2.128

**Published:** 2020-05

**Authors:** Khalil Rostami, Mohammad Ali Shahaboddin, Feizollah Niazi, Arian Karimi Rouzbahani, Sedigheh Nadri, Hormoz Mahmoudvand

**Affiliations:** 1 Department of Plastic and Reconstructive Surgery, Shahid Modares Educational Hospital, Shahid Beheshti University of Medical Sciences, Tehran, Iran;; 2 Student Research Committee, Lorestan University of Medical Sciences, Khorramabad, Iran;; 3 Department of Surgery, Lorestan University of Medical Sciences, Khorramabad, Iran

**Keywords:** Hirodoid, Ecchymosis, Edema, Periorbital, Eye, Rhinoplasty

## Abstract

**BACKGROUND:**

Several methods have been used to decrease the periorbital edema and ecchymosis after rhinoplasty. In this study, we evaluated the efficacy of hirudoid and dexamethasone in reduction of the periorbital edema and ecchymosis.

**METHODS:**

Sixty patients who underwent primary rhinoplasty were randomly divided into 3 groups. Group H received hirudoid cream, 3 times per day for 5 days from postoperative-day (POD). Group D received 10 mg of dexamethasone IV, immediately before surgery; and group C (control) received neither dexamethasone nor hirudoid. Two surgeons who were unaware of administered medications rated the severity of edema and intensity of ecchymosis, on 2^nd^, 5^th^, and 7^th^ POD.

**RESULTS:**

On 2^nd^ POD, the edema in group D was significantly lower than groups H and C; but there was no significant difference in severity and intensity of ecchymosis between 3 groups. On 7^th^ POD, the intensity of ecchymosis was significantly lower in group H in comparison to group C. When the difference between 2^nd^ and 7^th^ POD was evaluated, the resolution of severity of edema and intensity of ecchymosis was significantly better in group H (*p*<0.001).

**CONCLUSION:**

Hirudoid was shown to be effective in reducing edema and ecchymosis after rhinoplasty. The use of dexamethasone was effective in prevention of periorbital edema at early postoperative days, but it was not effective on resolution of ecchymosis.

## INTRODUCTION

Rhinoplasty as a widespread procedure in cosmetic surgery is performed on both soft and bony tissues and like other surgeries causes tissue traumas. Inflammatory responses to this trauma can result in morbidity of the patient, including ecchymosis and edema, which are prevalent and expectable outcomes in rhinoplasty.^1^ The most important cause of this complication is vascular trauma at the site of osteotomy.^2^ The extent and severity of these complications may be reduced by precisely observing surgical techniques, even they cannot be completely prevented.^2^^,^^3^

These complications can prolong the convalescence of patients and, at the same time, they are of interest to the patient, his/her relatives, and even to the physician, such that they sometimes frighten patients and their companions that would affect patient satisfaction. On the other hand, the more edema, the more adhesion would be. Also, the presence of ecchymosis (with greater intensity and extent) with prolonged discoloration causes post-inflammation hyperpigmentation (PIH), which can even create or intensify the black loop around the eye.^2^^,^^3^

In clinical trials, various methods have been employed trying to reduce these side effects, which are summarized as employment of steroids, adrenaline solution one per 100,000, ice bag, intravenous tranexamic acid, clonidine, herbal material such as Arnica, and topical use of vitamins K.^4^ Since the above agents are not impeccable either due to systemic effects or limitations in results of administration, if pharmacological agents can be used, which have easier and especially topical use and reduce effectively edema and ecchymosis, they can be routinely used in clinical settings.^4^^,^^5^

Hirodoid (heparinoid or HPS) has anti-clotting, fibrinolytic and anti-inflammatory effects, and until now, no study demonstrated the impact of hirodoid in reducing edema and ecchymosis following rhinoplasty, although other studies outside the field of surgery has been undertaken.^6^ Hirodoid cream has an active compound called mucopolysaccharide polysulfate (MPS) which is a semi-synthetic molecule produced by the sulfation of a mixture of glycosaminoglycan derived from mammalian cartilage, with an average molecular weight of about 9700 Dalton. Because of its chemical similarity to heparin, it is also called heparinoid. Hirodoids are used as an anti-coagulant, fibrinolytic and anti-inflammatory agent for clinical applications.^7^^,^^8^ The aim of this study was to investigate the therapeutic effect of this substance on edema and ecchymosis after rhinoplasty.

## MATERIALS AND METHODS

This study is a randomized prospective study approach, during which, rhinoplasty patients were randomly divided to three groups. The study was approved in the institution ethics committee. The study population consisted of all patients who underwent rhinoplasty surgeries by the authors at the Modarres and Khordad Health Centers and Sina Shemiran Limited Surgical Centers during the specified period. A written consent was provided from each patient. Based on the prepared questionnaire, patients’ information was recorded. In order to reduce the probability of error, facial photographs were taken on days 1^st^, 5^th^, and 7^th^ and edema and ecchymosis levels around their eyes were separately monitored and scored by two observers. 

The assessment was performed on primary septorhinoplasty patients undergoing osteotomy. The exclusion criteria were being diabetic or hypertensive, suffering from peptic ulcer, psychiatric patients, subjects with allergies to steroids and herodoids, women during menstruation or immediately before the menstrual phase, those who received anticoagulants and antiplatelet drugs. The operation was performed under general anesthesia by injecting epinephrine solution at one per 100,000 with a team of surgeons including three surgeons with the same surgical techniques and equipment, and the patients received similar postoperative care. 

Sixty patients aged between 18 and 45 years were enrolled and divided into three equal groups. Group D received a single dose of 10 mg dexamethasone intravenously just before surgery. Group H was treated with a topical hirodoid cream, three times daily for 3 days as a thin layer in the affected area 24 hours after surgery. Group C as the control group received no treatment, neither dexamethasone nor hirodoid. All patients underwent sub-general anesthesia. 

All patients received normal saline or ringer or ringer lactate at rate of 2 mL/kg/h during surgery and it was tried to keep the MBP in the range of 55-65. Patients whose blood pressure was not well controlled during the operation were excluded. Initially, 10 mL adrenaline solution was infiltrated at one per hundred thousand. Surgery was started after 10-15 minutes. Intra- and outer-nasal splint and nasal tampon were routinely used in all patients. The amount of intraoperative bleeding, duration of operation, and intraoperative complications were recorded. 

During the first 24 hours after surgery, the patients’ beds were inclined upward with 45 degrees. Intramuscular 25 mg pethidine, intravenous 1 gram apotel and 200 mL normal saline infusion for 20 minutes were administered to control pain. Patients received oral antibiotics for 5 days after surgery. Following the operations, patients were given acetaminophen codeine every 6 hours and 5 mg oxazepam tablets every 12 hours. The ice bag was also recommended for four days along with the head held high. 

**Fig. 1:**
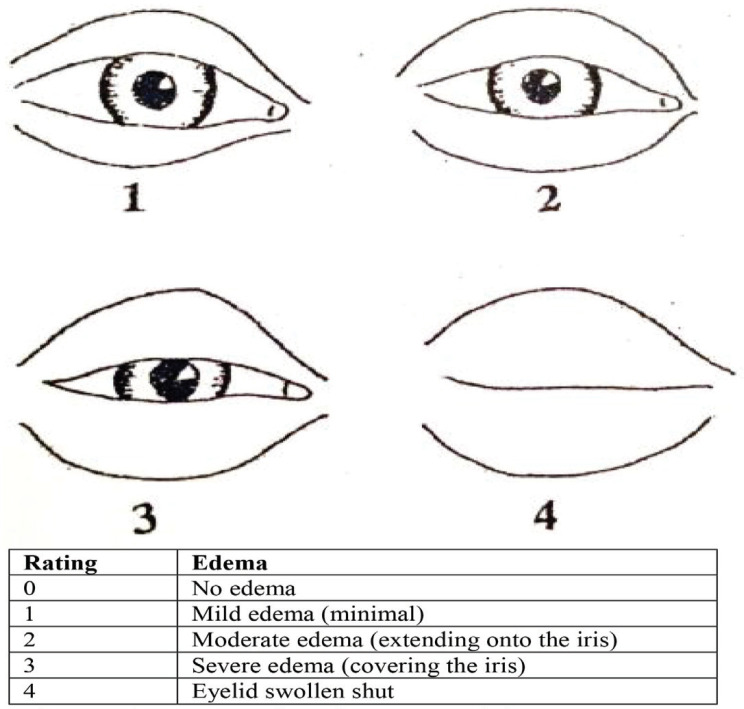
Severity scoring system for edema around the eye

**Fig. 2:**
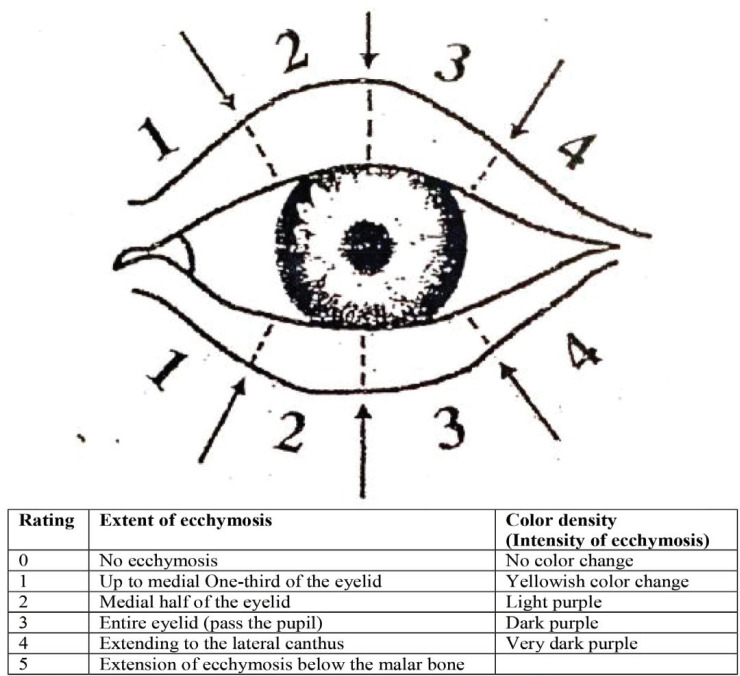
Severity and extent scoring system for ecchymosis around the eye

Patients and physicians were unaware of the nature of treatment and methodology until the end of the study. During the visit, the patients were photographed with a 10 megapixel resolution with a digital camera at a distance of 40 cm on the second, fifth and seventh days after surgery. Then, by two observers, severity of edema, and the severity and extent of ecchymosis were recorded in the questionnaire based on the scoring system for each patient, based on clinical and photographic findings (Figure 1 and 2). 

Statistical analysis of the study was performed using SPSS software (Version 15 for Windows, Chicago, IL, USA). Pearson’s Chi-square test was used to compare sex between groups. For statistical analysis, Kruskal-Wallis and Tamhane post-hoc tests were used to compare pair to pair the groups. In another part of the statistical comparison, the efficacy of each drug was compared to the control group. Since the variables were qualitatively ranked in terms of nature, statistical analysis was performed to compare them by three times using Friedman nonparametric test. Calculated values ​​(*p*<0.05) were considered statistically significant.

## RESULTS

In this study, 60 patients were enrolled (Table 1). Two patients were excluded from the study because of hypersensitivity, and the final analysis was performed on the remaining 18 patients. In the initial descriptive study in all the three groups, the severity and extent of edema and severity of ecchymosis at the first follow-up, that is the second postoperative day, were determined and presented in Table 2. In the dexamethasone group, the mean edema was numerically lower than the other two groups, while the difference was statistically significant (*p*<0.002). 

**Table 1:** Frequency of sex and mean age of patients in three groups

**Sex**	**Female**			**Male**		
**Group**	**Frequency**	**Mean**	**Standard** **deviation**	**Frequency**	**Mean**	**Standard** **deviation**
Dexamethasone	11	26.7	5.2	9	24.4	4.7
Hirodoid	13	26.8	7.5	5	28.4	3.0
Control	17	25.4	5.8	3	21.7	1.5
Total	41	26.2	6.1	17	25.1	4.4

There was no statistically significant difference between the three groups in terms of mean age (*p*>0.08).

**Table 2:** The status of patients in the three groups in terms of mean degree of edema, extent and severity of ecchymosis on the second day after surgery

**Group (no. of patients)**	**Degree of edema** **(Second day)**	**Extent of ecchymosis (Second day)**	**Severity of ecchymosis** **(Second day)**
Dexamethasone (20)	18.95	27.48	26.20
Hirodoid (18)	35.98	30.94	29.92
Control (20)	34.40	30.23	32.43
*p* value	*p*<0.002	*p*>0.7	*p*>0.4

The comparison between the three groups with Kruskal-Wallis nonparametric test was done and the results were mentioned in the text.

Comparing the two dexamethasone and hirodoid groups, as well as the two dexamethasone and control groups, it was found that the difference was significant (*p*<0.005), but there was no significant difference between the two groups of hirodoid and control (*p*>0.9). In addition, there was no significant difference between the three groups regarding the variables of the extent of ecchymosis and severity of ecchymosis on the second day after surgery. Comparison of other days of the study was performed for all three groups separately similar to the second day after surgery. Table 3 and 4 show the results of these comparisons.

**Table 3:** The status of patients in the three groups in terms of mean degree of edema, extent and severity of ecchymosis on the fifth day after surgery

Group (no. of patients)	**Degree of edema** **(Fifth day)**	**Extent** **of ecchymosis (Fifth day)**		**Severity of ecchymosis** **(Fifth day)**
Dexamethasone (20)	20.90	31.13	30.25	
Hirodoid (18)	29.17	25.67	21.58	
Control (20)	38..40	31.33	35.88	
*p* value	*p*<0.005	*p*>0.4	*p*<0.05	

**Table 4:** The status of patients in the three groups regarding the mean degree of severity of edema, extent and severity of ecchymosis on the seventh day after surgery

**Group (no. of patients)**	**Degree of edema** **(Seventh day)**	**Extent of ecchymosis (Seventh day)**	**Severity of ecchymosis** **(Seventh day)**
Dexamethasone (20)	24.23	31.65	31.68
Hirodoid (18)	26.69	23.42	21.11
Control (20)	37.30	32.83	34.88
*p* value	*p*<0.05	*p*>0.1	*p*<0.05

As shown in Table 3, this time on the fifth postoperative day, there was a significant difference between three study groups in terms of severity of edema and severity of ecchymosis, while the difference between dexamethasone and control groups was significant (*p*<0.005). Regarding severity of ecchymosis, the difference between the hirodoid and control groups was significant (*p*<0.02). For other groups, the difference was not statistically significant. Similar results were obtained for the seventh day when compared with the fifth day. 

As shown in Table 4, the severity of edema and severity of ecchymosis were significantly different in the three study groups, which was significant for the dexamethasone and control groups (*p*<0.01). In addition, for severity of ecchymosis, the difference between hirodoid and control groups was significant (*p*<0.05). The results of these comparisons did not show any statistically significant difference between the different groups for the extent of ecchymosis. However, in terms of mean degree, in the hirodoid group, both on day 5^th^ and on day 7^th^, the numerical value of this variable was lower than the other two groups, which was clinically significant, but not statistically significant. 

Perhaps the resulting difference in this complication may be clinically and statistically significant and valuable for patients by further studies on patients and careful evaluation and quantitative (not qualitative) measurement of the extent of ecchymosis. In another part of the statistical comparison, the efficacy of each drug was compared to the control group. Since the variables were qualitatively ranked in terms of nature, the necessary statistical analysis was performed to compare the three follow-ups by using Friedman nonparametric test, the results of which showed a decrease in the mean of edema severity in the groups of hirodoid and control. 

**Fig. 3:**
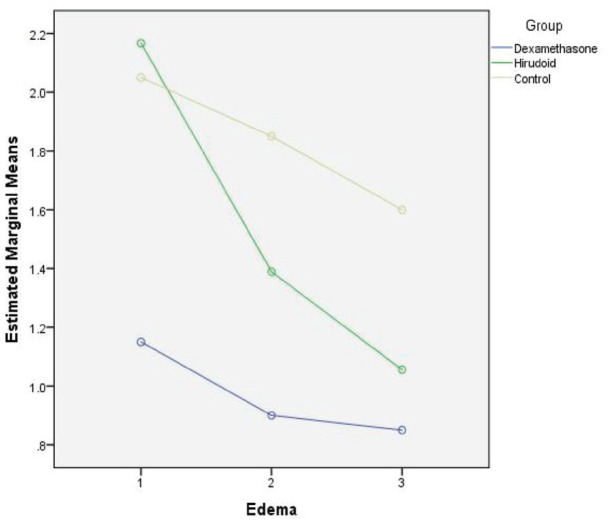
Comparison of three groups in three follow-up times in terms of change in severity of edema

**Fig. 4:**
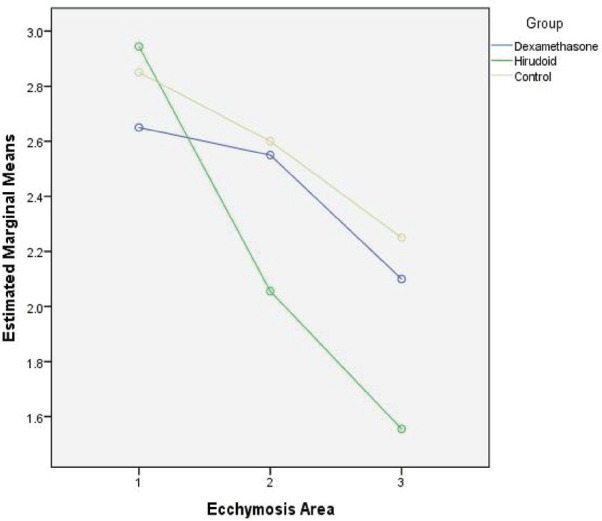
Comparison of three groups in three follow-up times in terms of change in ecchymosis extent

**Fig. 5:**
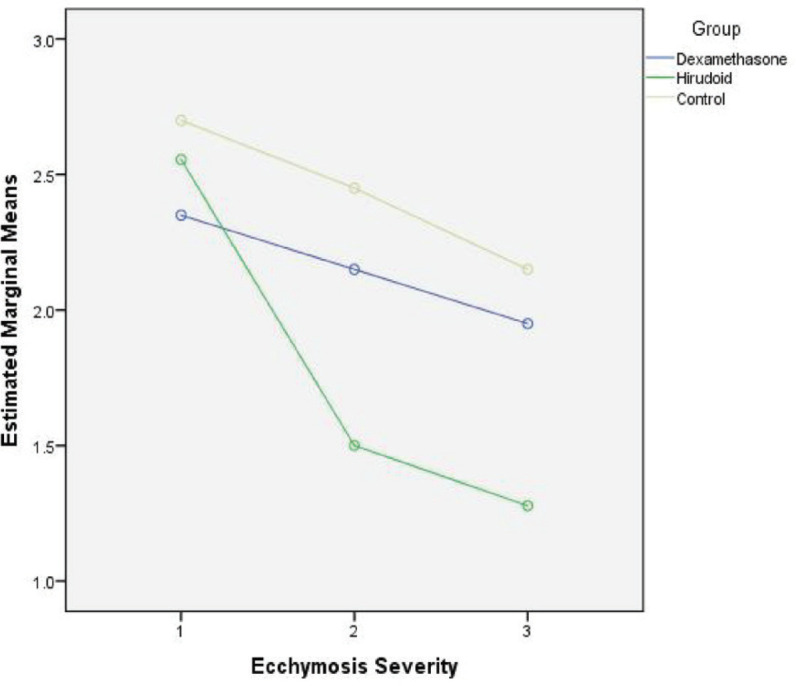
Comparison of three groups in three follow-up times in terms of ecchymosis severity

As can be seen in Table 5, the effect of dexamethasone on reducing ecchymosis extent was significant, but not significant in the other two variables between days 1^st^ and 5^th^, but the effect of hirodoid on all three variables was significant. Given that both the hirodoid and the control groups, a decrease in the severity of edema and ecchymosis and in the dexamethasone group was seen, and this was not confirmed, and the hypothesis that dexamethasone may not have a beneficial effect on the patient can be supported and reinforced. If no medication is taken too, the severity of edema and the extent and severity of ecchymosis are reduced spontaneously. Figures 3-5 showed the decreasing effect of the two drugs in comparison with the control group, at three follow-up times in the study, respectively, to be judged taking into account the statistical results mentioned in the tables. 

**Table 5:** The status of patients in the three groups in terms of mean degree of edema severity, extent and severity of ecchymosis in three postoperative examinations.

**Group (no. of patients)**	**Examinations**	**Degree of edema severity**	**Extent** **of ecchymosis**	**Severity of ecchymosis**
Dexamethasone (20)	Second Day	2.18	2.28	2.18
	Fifth Day	1.95	2.20	2.03
	Seventh Day	1.88	1.53	1.80
Statistical Result		*p*>0.15	*p*<0.005	*p*>0.28
Hirodoid (18)	Second Day	2.61	2.58	2.75
	Fifth Day	1.89	1.94	1.72
	Seventh Day	1.50	1.47	1.53
*p* value		*p*<0.001	*p*<0.001	*p*<0.001
Control (20)	Second Day	2.30	2.30	2.35
	Fifth Day	2.03	2.05	2.03
	Seventh Day	1.68	1.65	1.63
*p* value		*p*<0.05	*p*<0.05	*p*<0.01

## DISCUSSION

The results of this study showed that the consumption of hirodoid had a significant effect on accelerating the removal of edema and the extent and severity of periorbital area of ecchymosis in patients undergoing primary rhinoplasty. In this study, there were no serious complications following the use of hirodoid, and only two patients were diagnosed with contact dermatitis due to inflammation and itching. The investigations also showed that preoperative dexamethasone had a positive effect on the severity of edema in patients with a relatively good and statistically significant degree.^9^

It was despite the fact that the severity of edema in the patients receiving hirodoid was initially similar to that of the control group, but by starting the use of hirodoid, the rate of edema decreased very rapidly and became closer to dexamethasone on day 7^th^. It should be noted that the effect of dexamethasone in decreasing edema is related to day 1^st^ and in preventing edema, but after edema, its gradual elimination rate during the days 2^nd^ to 7^th^ in the dexamethasone group was not significantly different from the control group.

Statistical results also showed that in patients receiving dexamethasone, the severity of ecchymosis did not differ significantly between days 2^nd^ and 7^th^, while the extent of ecchymosis decreased significantly. This is not clinically relevant because, as can be seen in Table 5, a significant decrease in the severity and extent of ecchymosis was observed in days 2^nd^ to 7^th^ in both the control and the hirodoid groups. The rate of severity and extent of ecchymosis in the hirodoid group was significantly higher than that of the dexamethasone and control groups.

It should be noted that the results could not indicate an observer error, because the evaluation of this variable and determination of the degree of ecchymosis were performed by different observers with the patient’s facial pictures and none of the observers were aware of the patient group. The results are consistent with other researchers showing that pre-orbital edema and ecchymosis in patients who received a single dose of methylprednisolone before surgery were significantly lower than the control group.^10^

Similar results were demonstrated in the first two days in patients receiving a single dose of dexamethasone preoperatively.^11^ The current study showed only a decrease in the severity of edema on the second day after operation in patients receiving a single dose of dexamethasone, but no decrease in postoperative ecchymosis in dexamethasone group. In terms of severity and extent of ecchymosis, it was found that dexamethasone injection had no effect on increasing or decreasing the severity and extent of ecchymosis around the eye, and on the other hand, it only affected removing rate of the extent of ecchymosis, which of course this effect was lower than hirodoid.^12^

This is in contrast to the results of other researchers^12^ showing that dexamethasone injection slowed the rate of recovery of ecchymosis’ extent and severity. Another study on Arnica showed that dexamethasone was effective in reducing edema following rhinoplasty, but had no effect on reducing ecchymosis. However, our study showed that hirodoid cream was effective in reducing both edema and the severity and extent of ecchymosis around the eye.^12^

The results of our study indicated that the use of hirodoid cream was a simple and low impact method to reduce the rate of edema, severity and extent of ecchymosis after rhinoplasty operation, which may result in physical, psychological, social and economic effects and patient’s satisfaction. Preoperative dexamethasone injection, although initially preventing peripheral edema, had no effect on reducing the severity and extent of ecchymosis after rhinoplasty. Given the above, the use of topical post-rhinoplasty hirodoid cream to reduce edema and ecchymosis around the eyes seems reasonable and increased patient satisfaction.

Taken together, the results of our study suggested that it is likely that a combination of two therapies, namely intravenous dexamethasone injection before rhinoplasty as well as post-operative topical administration of hirodoid cream, can reduce the complications of edema and ecchymosis around the eye, and can have a significant therapeutic efficacy and can increase patient’s satisfaction.
